# Vesicle budding caused by lysolipid-induced asymmetry stress

**DOI:** 10.1016/j.bpj.2023.08.023

**Published:** 2023-08-29

**Authors:** Lisa Hua, Michael Kaiser, Iulia Carabadjac, Annette Meister, Gerd Hause, Heiko Heerklotz

**Affiliations:** 1 Institute of Pharmaceutical Sciences, University of Freiburg, Freiburg, Germany; 2 ZIK HALOmem and Institute of Biochemistry and Biotechnology, MLU Halle-Wittenberg, Halle, Germany; 3 Biozentrum, MLU Halle-Wittenberg, Halle, Germany; 4 Leslie Dan Faculty of Pharmacy, University of Toronto, Toronto, Canada; 5 Signaling Research Center BIOSS, University of Freiburg, Freiburg, Germany

## Abstract

Lysolipids such as lauroyl, myristoyl, and palmitoyl lysophosphatidylcholine (LPC) insert into the outer leaflet of liposomes but do not flip to the inner leaflet over many hours. This way, they create asymmetry stress between the intrinsic areas of the two leaflets. We have studied how this stress is relaxed with particular emphasis on the budding and fission of small (diameter 20–30 nm) daughter vesicles (DVs). Asymmetric flow field-flow fractionation was utilized to quantify the extent of budding from large unilamellar vesicles after exposure to LPC. Budding starts at a low threshold of the order of 2 mol% LPC in the outer (and ≈0 mol% LPC in the inner) leaflet. We see reason to assume that the fractional fluorescence intensity from DVs is a good approximation for the fraction of membrane lipid, POPC, transferred into DVs. Accordingly, budding starts with a “budding power” of ≈6 POPC molecules budding off per LPC added, corresponding to a more than 10-fold accumulation of LPC in the outer leaflet of DVs to ≈24 mol%. As long as budding is possible, little strain is built up in the membranes, a claim supported by the lack of changes in limiting fluorescence anisotropy, rotational correlation time, and fluorescence lifetime of symmetrically and asymmetrically inserted TMA-DPH. At physiological osmolarity, budding is typically limited to 20–30% of budded fraction with some batch-to-batch variation, but independent of the LPC species. We hypothesize that the budding limit is determined by the excess area of the liposomes upon preparation, which is then used up upon budding given the larger area-to-volume ratio of smaller liposomes. As the mother vesicles approach ideal spheres, budding must stop. This is qualitatively supported by increased and decreased budding limits of osmotically predeflated and preinflated vesicles, respectively.

## Graphical abstract

**Figure:**
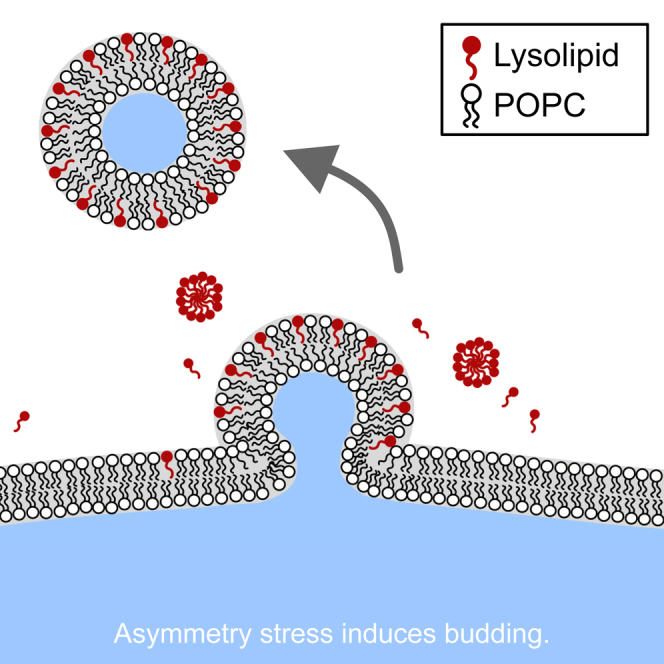

## Significance

The asymmetric incorporation of compounds into the lipid membrane causes asymmetry stress, which is involved in membrane remodeling processes and is discussed as a mode of action of antimicrobial peptides. Here, we studied vesicle budding and fission as one out of several mechanisms of asymmetry stress relaxation. We quantify, to our knowledge for the first time, three key parameters of additive-induced budding: threshold asymmetry, budding activity/power, and budding limit. This offers an answer to the question of which relaxation mechanism actually takes place: budding has a lower threshold than other mechanisms and keeps asymmetry stress very low but reaches its limit as the excess area of the liposomes is “used up.” Then, other mechanisms have to kick in.

## Introduction

Lysophosphatidylcholines (LPCs) are metabolites of phospholipids where the *sn*-2 acyl chain has been cleaved off by phospholipase A2. With only a single acyl chain left, lysolipids can form micelles. LPCs occur in the human body mainly bound to human serum albumin or to low-density lipoproteins in the plasma (1,2). A few LPC molecules are also found in the membrane. The physiological functions of LPC are still not fully understood but it is, for example, increasingly recognized as a key marker with cardiovascular and neurodegenerative diseases (2). Cancer may be accompanied by decreased LPC plasma levels (3). It is assumed that LPC can act as an agonist on several G-protein-coupled receptors involved in angiogenesis (4) and chemotaxis (5). Besides being a signaling molecule, LPC is also known for its contribution to lipid membrane remodeling processes. It was observed that lysolipids can inhibit fusion processes (6) and alter the mechanical properties of the lipid bilayer, which modulate the channel function of gramicidin (7). Some of these physiological effects and functions are likely related to the detergent-like behavior of LPCs.

Interactions between detergents and membranes have been thoroughly studied (8). Typical detergents are amphiphiles with an inverted cone shape that prefer convex aggregate surfaces as in micelles and induce positive monolayer curvature stress when inserted into a lipid leaflet. The well-known three-stage model (9,10) describes the interactions of liposomes with detergents with a high flip-flop rate. The saturation boundary marks the maximum detergent-to-lipid ratio without micelle formation, whereas the solubilization boundary represents the minimum detergent to lipid ratio in mixed micelles without bilayer formation.

**Figure 1:**
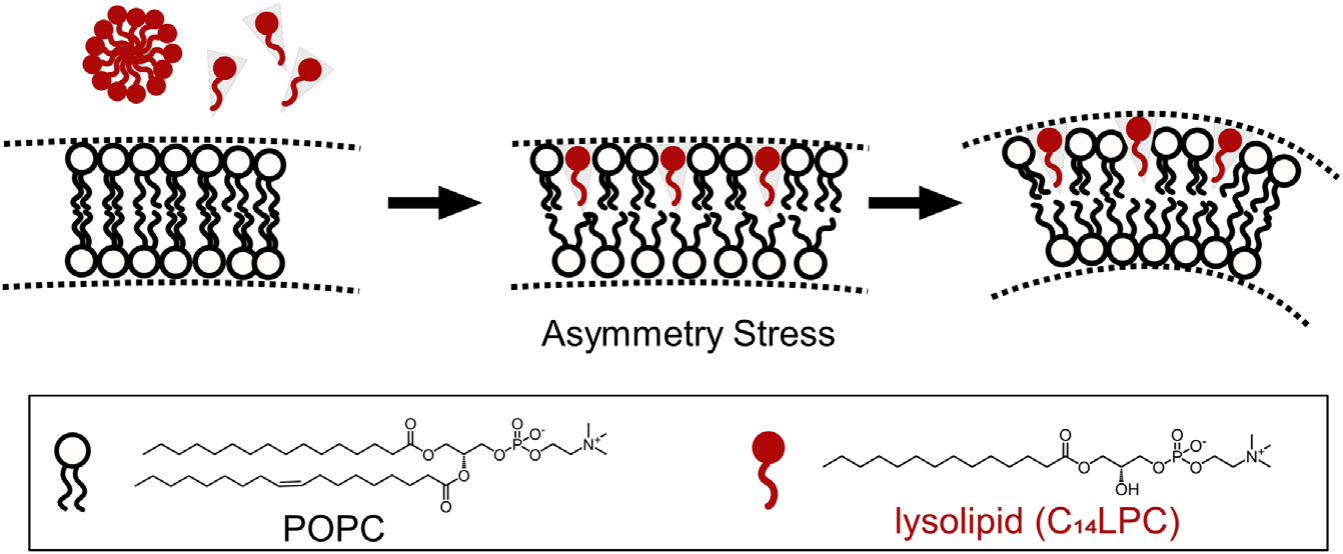
Schematic drawing of the asymmetric insertion of lysolipids (*red*) into a POPC bilayer (*black*). The asymmetric distribution of lysolipids in the outer leaflet causes a spontaneous curvature of the bilayer. To see this figure in color, go online.

However, the three-stage model is not applicable to detergents like LPC that display a slow transbilayer translocation rate because, kinetically, the bilayer-to-micelle transition of a membrane requires detergent molecules to reside in both leaflets (11). Added to a liposomal dispersion, such detergents insert selectively into the outer leaflet of liposomal membranes and expand its intrinsic area asymmetrically (12). The result is asymmetry stress, also referred to as “bilayer curvature stress” or “differential stress,” representing a tendency of the bilayer to bend as a “bilayer couple” (13). The process is illustrated in a schematic drawing in Fig. 1.

The primary consequence of asymmetry stress is the lateral stretching of the lipids in the underpopulated (here: inner) leaflet and a compression of those in the overpopulated one. When the energy stored in this stress reaches the activation energy of a relaxation mechanism, the stress will be relaxed or at least limited. Different mechanisms have been reported or hypothesized, referred to as 1) budding or exovesiculation (14,15), 2) staying out (16,17), 3) micellar solubilization (11), 4) cracking in (12,18), 5) stimulated flip-flop (19), and 6) shedding (20). First, the budding and exovesiculation of very small daughter vesicles (DVs) relaxes asymmetry stress because, given their size and extreme curvature, budded vesicles have a larger area of the outer compared with the inner leaflet. The budding effect of LPC has been reported for giant unilamellar vesicles (GUVs) (21,22,23,24,25), but could, to our knowledge, not be quantified yet. To quantify budding with GUVs, this system would require finely tuning the vesicle size, which is not feasible with current oil-free preparation techniques. Second, the energy inherent in the stress may supersede, at some membrane content, the energy gain of membrane insertion of the stress-inducing agent. This has also been demonstrated for C_12_LPC (17) and digitonin (16), which then stay out of the membrane and accumulate in the aqueous solution so that the stress does not increase any further. Third, as the surfactant staying out of the membrane reaches its critical micelle concentration (CMC) in solution, it will form micelles that then, with kinetics of many hours or days, capture lipids from the outer leaflet, relax stress, and ultimately cause what has been termed “micellar solubilization” of the whole membrane (26). Fourth, a pathway alternatively to “staying out” was referred to as “cracking in”—relaxing asymmetry stress by a small and transient membrane defect that allows for lipid and surfactant to flip to the inner leaflet and may be accompanied by transient membrane leakage also for aqueous solutes. This requires surfactants with higher insertion force, including alkyl maltosides (12) and amphiphilic (lipo)peptides such as surfactin (27), pseudodesmin (28), viscosin (29), and tolaasin (30). A fifth mechanism, also reported for dodecylmaltoside (19), is the acceleration of the otherwise very slow flip-flop kinetics of the lipids to balance asymmetry. Finally, the shedding of mixed micelles by exvagination from the outer membrane leaflet has been hypothesized as another plausible relaxation mechanism (20).

The puzzling question is, which of these potential relaxation mechanisms or, to be more precise, which combination thereof, is actually pursued by the system in a given case. LPCs were shown to induce budding from GUVs and staying-out and micellar solubilization from large unilamellar vesicles (LUVs) (17). Is budding also occurring from LUVs and if so, why does it not render the staying-out pathway unnecessary? The aim of this study is to answer these questions and this way, contribute both to the better understanding of LPC effects on membranes and, more generally, of the mechanisms to relax asymmetry stress.

Vesicle budding is a process that is also involved in multiple biological processes. In eukaryotic cells, vesicle budding is required for the intracellular membrane transport between organelles (31), but also in synapses between neurons (32). It also takes place during virus budding (33) and is also needed for asexual reproduction of microbes and therefore a possibility to study the origin of cellular life (34,35). Besides the already known protein-centered mechanisms to induce membrane curvature (36,37,38), the asymmetric incorporation of LPC can contribute to or modulate the overall membrane remodeling process (39). In addition, asymmetry stress not only induces or opposes shape changes but is also discussed as mode of action for antimicrobial peptides (28,29,30).

In our experiments, we used LUVs to study the effect of LPC. By using asymmetric flow field-flow fractionation (AF4), we were able to establish an assay to quantify the budded fraction. Using LUVs also provides the advantage that data typically generated in LUV systems, such as partition coefficients (17,40,41) and leakage data, are applicable to our experiments. By studying the parameters influencing the asymmetry-stress-induced budding process, we can understand how the membrane deals with asymmetry stress. The results can potentially be transferred to other membrane-impermeant amphiphiles with similar properties as LPC, such as digitonin (16) or gangliosides (42,43).

## Materials and methods

### Materials

1-Palmitoyl-2-oleoyl-*sn*-glycero-3-phosphocholine (POPC) was kindly provided by Lipoid (Ludwigshafen, Germany). 1-Lauroyl-2-hydroxy-*sn*-glycero-3-phosphocholine (C_12_LPC), 1- myristoyl-2-hydroxy-*sn*-glycero-3-phosphocholine (C_14_LPC), 1-palmitoyl-2-hydroxy-*sn*-glycero-3-phosphocholine (C_16_LPC), and 1,2-distearoyl-*sn*-glycero-3-phosphoethanolamine-*N*-(7-nitro-2-1,3-benzoxadiazol-4-yl) ammonium salt (NBD-DSPE) were purchased from Avanti Polar Lipids (Alabaster, USA). 1,6-Diphenyl-1,3,5-hexatrien-4′-trimethylammonium-tosylat (TMA-DPH), Tris(hydroxymethyl)-aminomethane (Tris), sodium chloride (NaCl), and sodium azide (NaN_3_) were purchased from Sigma-Aldrich-Chemie (Taufkirchen, Germnay). Ultrapure water was obtained in-house from an arium pro system from Sartorius AG (Göttingen, Germay). All other chemicals were purchased from Carl Roth (Karlsruhe, Germany) and were of analytical grade.

### Vesicle preparation

Fluorescence-labeled and pure POPC liposomes were prepared by thin-film hydration and extrusion (44). POPC and NBD-DSPE dissolved in chloroform were combined to obtain a fraction of 1 mol% of fluorescent lipid for AF4 experiments. For time-resolved anisotropy measurements with symmetric localization of TMA-DPH, either 1 or 0.1 mol% of TMA_DPH were added to POPC in chloroform. The solutions were dried under vacuum overnight. The film was rehydrated with buffer (10 mM Tris, 100 mM NaCl, 0.02% NaN_3_ [pH 7.4] at 25°C). After five freeze-thaw cycles, the lipid dispersion was extruded 10 times through two stacked polycarbonate membranes from Whatman (Buckinghamshire, UK) of pore size 80 nm using a Lipex thermobarrel extruder by Evonik (Essen, Germany) at 25°C using nitrogen at a pressure of 20 bar. The intensity-weighted hydrodynamic diameter (Z-Avg. (*d*_H_)) of the large unilamellar vesicles (LUVs) was confirmed by dynamic light scattering (DLS) to be around 100 nm and the polydispersity index (PDI) to be ≤0.1.

### DLS

DLS was performed at 25°C on a Nano-ZS Zetasizer by Malvern Panalytical (Kassel, Germany) equipped with a 633 nm He-Ne laser at a detection angle of 173°. Data acquisition was performed with Zetasizer software (v.7.13); viscosity and refractive index of the medium were calculated from the software’s database. Attenuator and measurement position were optimized by the software automatically. The same software was used to obtain size and size distribution.

### Determination of lipid concentration

Lipid concentrations of LUVs and LPC dispersion were determined by Bartlett assay (45).

### Incubation of LUVs with LPC

C_12_/C_13_/C_14_LPC stock solutions were prepared in the same buffer as the vesicles (10 mM Tris, 100 mM NaCl, 0.02% NaN_3_ [pH 7.4] at 25°C). The maximal concentration of the stock solution did not exceed 45 mM. The POPC liposomes with 1 mol % NBD-DSPE were incubated at room temperature (20 ± 2°C) with the respective LPC concentration. The incubation time of all experiments shown in the main text was approximately 2 min before analysis on the AF4. Longer incubation times were only used to validate the method as presented in the supporting material.

### AF4

Particles were separated based on size by AF4 using an Eclipse flow controller by Wyatt Technologies (Dernbach, Germay) controlled by VISION RUN (v.3.0.1.12). Separations took place in an SC separation channel (Wyatt Technologies) equipped with a W490 spacer (Wyatt Technologies) and 10 kDa regenerated cellulose membrane (Wyatt Technologies). Coupled to the channel were multiangle laser light scattering (MALS) detector and the fluorescence spectrometer for online detection. Separations were performed with 5 *μ*L samples at 3.5 mM POPC concentration. A detailed elution profile is given in the supporting material. The separation on the AF4 was executed at room temperature (20 ± 2°C).

### MALS

Online MALS measurements were conducted on a DAWN HELEOS II (Wyatt Technologies) equipped with a 662 nm Ga-As laser used at full intensity. Data acquisition was performed with VISION RUN (v.3.0.1.12) and ASTRA (v.8.0.1.21). Data analysis was performed with ASTRA using detectors 4 (38°) through 18 (147°). To determine geometric size of vesicles, the coated sphere model (46) was employed; a refractive index of 1.333 was used for the medium and a shell thickness and refractive index of 3.7 nm (47) and 1.450 (48), respectively, for the vesicles.

### Determination of the fraction of fluorescence intensity originating from DVs, *X*_F_^DV^

Online fluorescence detection was performed on a 1260 Infinity Fluorescence Detector by Agilent Technologies (Waldbronn, Germany) with excitation and emission wavelengths of 460 and 520 nm, respectively. Due to properties inherent to the investigated system, elution peaks were not baseline separated. Instead of directly integrating peaks, the chromatograms were deconstructed to approximate the extent of budding. A detailed explanation of this deconstruction is given in the supporting material.

### Time-resolved anisotropy of TMA-DPH

The samples were composed of 0.5 mM POPC LUVs with 0.1 or 1 mol% TMA-DPH content and contained 1.7 vol % methanol in addition to the buffer (10 mM Tris, 100 mM NaCl, 0.02% NaN_3_ [pH 7.4] at 25°C). For the experiments with TMA-DPH inserted symmetrically in both leaflets of POPC LUVs, vesicles were prepared as described above. For TMA-DPH inserted in the outer leaflet only, a methanolic solution of TMA-DPH was added to POPC LUV dispersion in buffer. After an incubation for a minimum of 2 h at 25°C, stirring at 400 rpm, C_12_LPC was added to the sample and incubated for 10 min. Measurements of time-resolved anisotropy were executed with the high-performance spectrometer FluoTime 300 by PicoQuant (Berlin, Germany) in quartz cuvettes by Hellma (Müllheim, Germany) (optical path of 10 × 10 mm) under continuous stirring at 25°C. Measurement setup and the initial analysis of the data were carried out with EasyTau Software (2.2.3293). Excitation was performed at a wavelength of 355 nm with laser polarization of 0° at a frequency of 16.67 MHz, laser intensity of 7.2, and pulse width of 25 ps. Emission was recorded through a 355 nm long-pass filter at 430 nm with detection band pass of 5 nm with the emission polarizer set to 0, 54.7, and 90°, respectively. *G*-Factor was recorded with the same instrumental setup with laser polarization at 90° and calculated by manual alignment of emission decays at polarization of 0 and 90°. Calculation of limiting anisotropy (*r∞*) was carried out assuming a mono-exponential decay, using a tail fit. The goodness of the fit was evaluated by the means of reduced χ^2^ (maximum of 1.3 at high LPC concentrations) and bootstrap error analysis; neither shown.

### Cryo-TEM

Vitrified specimens for cryo-TEM were prepared by a blotting procedure, performed in a chamber with controlled temperature and humidity using a LEICA grid plunger. A drop of the sample suspension (1 mg mL^−1^) was placed onto an EM grid coated with a holey carbon film (C-flat, Protochips, Morrisville, NC). Excess solution was then removed with a filter paper, leaving a thin film of the solution spanning the holes of the carbon film on the EM grid. Vitrification of the thin film was achieved by rapid plunging of the grid into liquid ethane held just above its freezing point. The vitrified specimen was kept below 108 K during storage, transfers to the microscope, and investigation. Specimens were examined with a LIBRA 120 PLUS instrument by Carl Zeiss Microscopy (Oberkochen, Germany), operating at 120 kV. The microscope is equipped with a Gatan 626 cryotransfer system. Images were taken with a BM-2k-120 Dual-Speed on axis SSCCD-camera by TRS (Dünzelbach, Germany). All samples for cryo-TEM were incubated 1 h at room temperature or 65°C as specified in the figure.

## Results

### Cryo-TEM shows DVs formed by lysolipid-induced budding from LUVs

**Figure 2:**
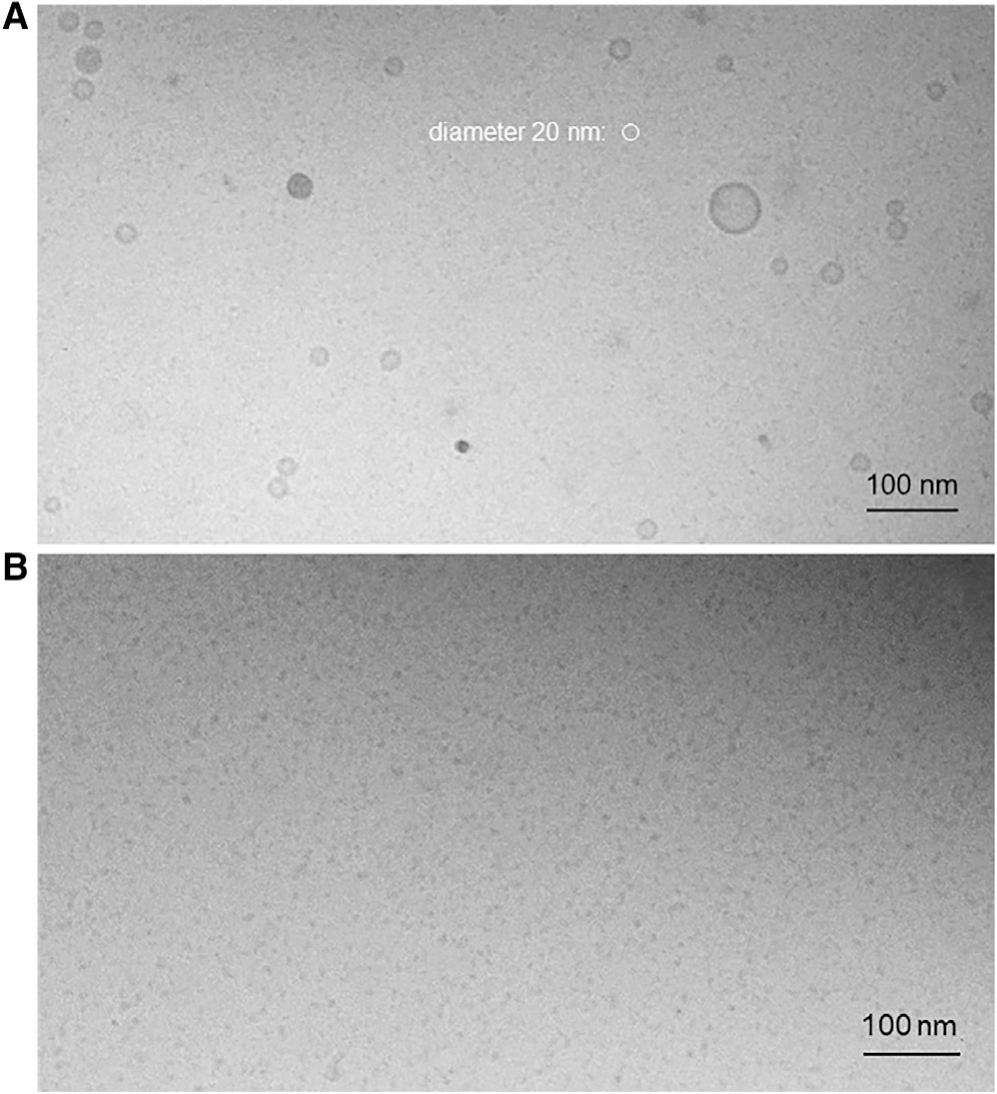
Cryo-TEM images of a sample of 5 mM POPC LUV exposed to 5 mM C_12_LPC. (*A*) Shows small daughter vesicles with sizes of the order of 20 nm (note white circle as a representation corresponding to 20 nm diameter for comparison). (*B*) Shows the same sample after heating it up to 65°C and cooling it down again to promote LPC flip-flop between the outer and inner leaflet. After temperature-induced translocation of LPC to the inner leaflet of the vesicles, the kinetic barrier to solubilization had vanished and all vesicles were disintegrated to mixed micelles. For reference pictures taken in the absence of LPC, see Fig. S2.

The qualitative identification of the small particles induced by the addition of C_12_LPC to LUV dispersions (see below) to be very small vesicles was done by cryo-TEM. Fig. 2 shows a picture of a dispersion of 2 mM POPC LUV exposed to 5 mM C_12_LPC. A small white circle demonstrates the size of a sphere with a diameter of 20 nm, showing that such very small lipid vesicles are abundant in the sample. We interpret these very small vesicles, which are not formed in the absence of LPC (see supporting material for reference), to be DVs having budded off from the original LUVs. At the same time, some larger vesicles persist, referred to as mother vesicles (MVs).

Fig. 2*B* shows the same sample as in Fig. 2 *A* but after heating it up to 65°C and cooling it down to room temperature again. In line with conclusions from other methods and for similar systems (17,49), this heat treatment has allowed for flipping of LPC from the outer to the inner leaflet resulting in a complete dissolution of all vesicles to mixed micelles. As expected given the maximal length of a C_12_LPC molecule of less than 2 nm, these micelles are considerably smaller than the DVs seen before heat treatment (Fig. 2 *A*). Furthermore, micelles lack the contrast between the surface and core that is seen for DVs.

The dramatic effect of the heat treatment illustrates again that the vesicles seen in Fig. 2 *A* are nonequilibrium structures that are kinetically stabilized merely by the kinetic barrier to LPC insertion into the inner leaflet. If the POPC LUV sample is heated up without addition of LPC, the vesicles stay intact (see Fig. S2).

Interestingly, Stuart and Boekma (20) discuss micelle shedding and not budding, but their Fig. 3 *D* shows tiny vesicles, suggesting that also dodecyl maltoside, another membrane-impermeant detergent, induces vesicle budding before solubilization.

### DVs can be detected and sized after separation via AF4

AF4 separates particles with respect to their hydrodynamic size and permits characterizing them by light scattering and fluorescence intensity. To obtain a signal in the fluorescence detector, 1 mol% of NBD-labeled lipid was included in the preparation of the vesicles. The geometric radius was obtained from the angle-dependent light scattering intensity.

**Figure 3:**
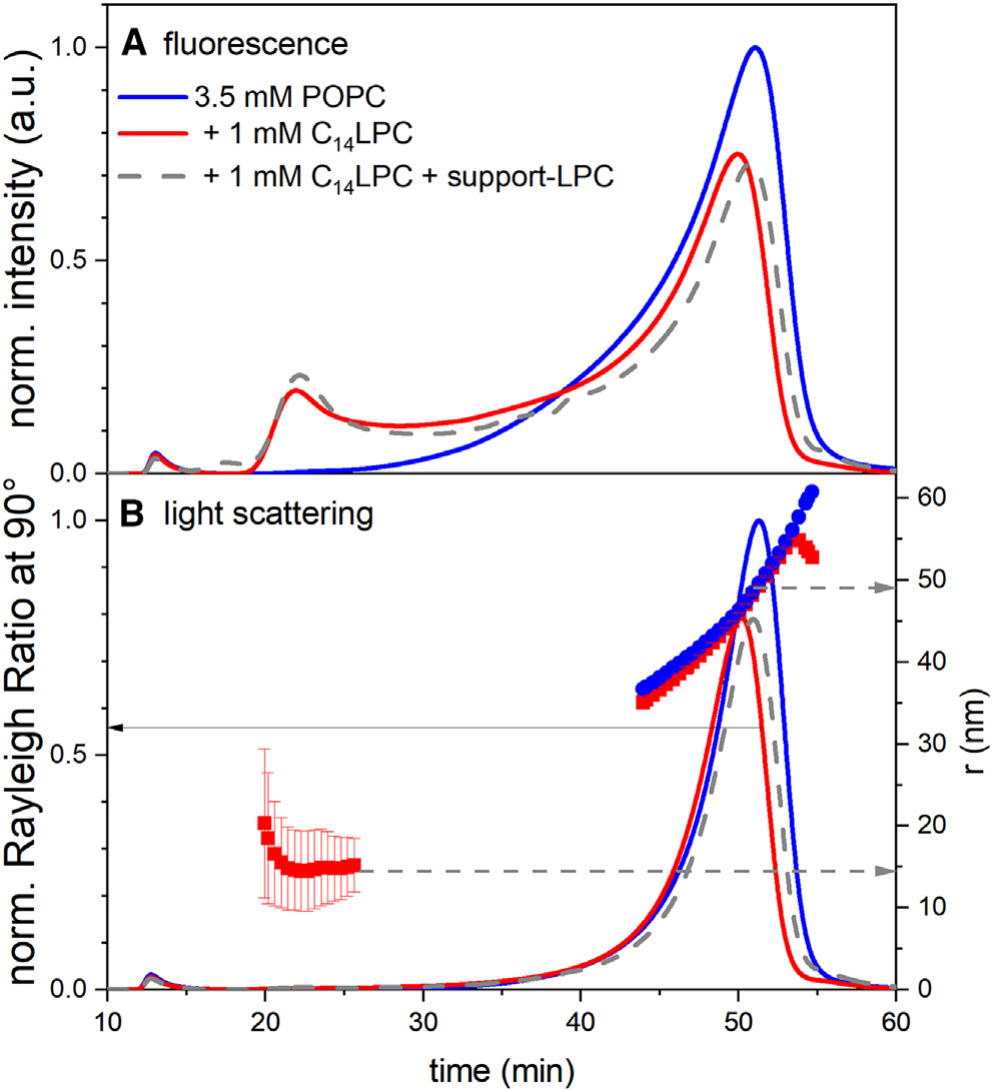
Normalized AF4 chromatograms obtained by fluorescence (*A*) and multiangle light scattering (MALS, *B*), the latter yielding intensity (*B*, left axis, *curves*) and radius (*B*, right axis, *symbols*), all as a function of elution time in the AF4. The graphs refer to fluorescence-labeled POPC LUVs incubated with 1 mM C_14_LPC (*red solid line*, *red squares*) and without addition of LPC (*blue solid line*, *blue dots*). Error bars represent the standard error. The gray dashed line represents a repetition of the experiment with LPC, now with 0.035 mM support-LPC in the elution buffer. Note that the MALS signal (*B*) scales with the sixth power of particle radius but fluorescence scales linearly with (labeled) lipid content. To see this figure in color, go online.

Freshly extruded vesicles without LPC treatment eluted between 45 and 55 min and showed an intensity-weighted average radius of 49 nm (*blue lines* in Fig. 3 *A* and *blue crosses* in Fig. 3 *B*, *right ordinate*). This is in line with the measured hydrodynamic radius, which was determined as 49 nm in DLS.

The intensity-based size distribution in Fig. 3 *B* is somewhat asymmetric, including a significant fraction of particles with shorter elution times (down to about 30 min). Since the intensity of scattered light increases with the sixth power of the radius, the amount of lipid in these smaller vesicles is underrepresented in Fig. 3 *B*. This is illustrated by the fluorescence intensity (Fig. 3 *A*), which should be proportional to the amount of lipid in a certain size fraction if the labeled lipid distributes homogeneously. Note that no fluorescence can be detected at elution times below 30 min for freshly extruded POPC vesicles without addition of LPC.

By adding LPC to the POPC vesicles before starting the AF4 experiment, the profile changes dramatically (*bold red line* in Fig. 3 *A*). A novel vesicle fraction is created that starts to elute after about 20 min and has an intensity-weighted radius of *r* ≈ 15 nm (*red squares* in Fig. 3 *B*). This fraction can be assigned to the DVs arising from lysolipid-induced budding and fission. This process is not accompanied with leakage of the interior volume as shown in leakage experiments (see Figs. S4 and S5, (50)). This supports the findings of cryo-TEM (Fig 2) proposing a mechanism of budding off of DVs rather than the pinching off of mixed micelles from closed vesicles (20).

It should be emphasized that small unilamellar vesicles produced by strong shear forces by sonication or otherwise can hardly be as small as the 20 nm or even less as observed here. Tearing apart small patches of bilayer that are then forced by their hydrophobic edges to close to vesicles involves strong bending of an intrinsically planar bilayer. The resulting bending stress is relaxed by fast fusion to produce SUV of the order of 30 nm that are kinetically stable for a few hours before growing even bigger. LPC-induced DVs are fundamentally different. Their asymmetric bilayer has a strongly positive intrinsic curvature, because it contains more molecules in the outer than the inner leaflet in the first place. In addition, as discussed in detail below, their outer, LPC-enriched leaflet shows a strongly positive monolayer curvature. Both effects together make the membrane bend spontaneously to very small but largely curvature stress-free vesicles that do not tend to fuse as long as the LPC remains in place.

### Dilution effects due to AF4 separation are reduced by addition of “support”-C_14_LPC in the AF4 eluent

As seen in Fig. 3 *A*, the daughter and MV peaks are not baseline-separated in the chromatogram and an intermediate population between the two peaks is apparent. It appears that DVs show a broad, asymmetric size distribution with the majority of lipid in vesicles of the order of 30 nm in diameter but also a shoulder reaching to larger sizes. This might be a consequence of the heterogeneous or varying conditions for the budding process at different points in volume or in time. An alternative explanation for the larger, budding-induced vesicles is a refusion of originally formed, 30 nm vesicles during the separation process. Since the sample is strongly diluted by the elution buffer during separation, LPC must be considered to be extracted from the DVs so that these should become unstable and may fuse with each other. The latter hypothesis was tested by adding LPC into the AF4 eluent so that the release of LPC out of the membrane shall be reduced (*gray dashed line* in Fig. 3 *A*). The concentration of “support-LPC” in the flow buffer was 0.035 mM, somewhat below the CMC of 0.045 mM (51) and lower than the concentration used to originally induce budding. Indeed, the small daughter peak at 20 min is more defined and the intermediate size signal was reduced, supporting the hypothesis of refusion, but complete baseline separation between the daughter and mother peaks could not be achieved.

### Budded fraction, *X*_F_^DV^, is estimated in terms of integrated fluorescence of novel, small vesicles

The total fluorescence integral in the AF4 chromatograms with and without LPC stays the same (see Fig. S3), showing that the quantum yield of NBD-DSPE does not change upon budding. This, in turn, implies that the fraction of the fluorescence intensity arising from DVs, *X*_F_^DV^, agrees essentially with the fraction of NBD-DSPE residing in DVs.

The fluorescence was assigned to either DVs or MVs to quantify the budded fraction. The detailed procedure is explained in the supporting material. In brief, the blank curve recorded without LPC (*blue line* in Fig. 3) was fitted to the large-size region of the fluorescence profile by a shift along the time axis and proportional stretching on the intensity axis. The resulting curve was assumed to approximate the contribution of the fluorescence arising from MVs and subtracted from the chromatogram after budding (*red line* in Fig. 3 *A*) to estimate the contribution of DVs to the overall chromatogram. The integral of the normalized fluorescence intensity *F(t)* of the DVs divided by the overall integral of *F(t)* then defined the budded fraction of fluorescence, *X*_F_^DV^. The standard incubation time of 2 min of LPC with the vesicles before AF4 separation appears to suffice for the system to reach a steady state. Within up to 11 h of incubation, the shape of the chromatograms and the resulting *X*_F_^DV^ remain stable (see Fig. S6).

### Budding starts at roughly 2% asymmetry threshold

**Figure 4:**
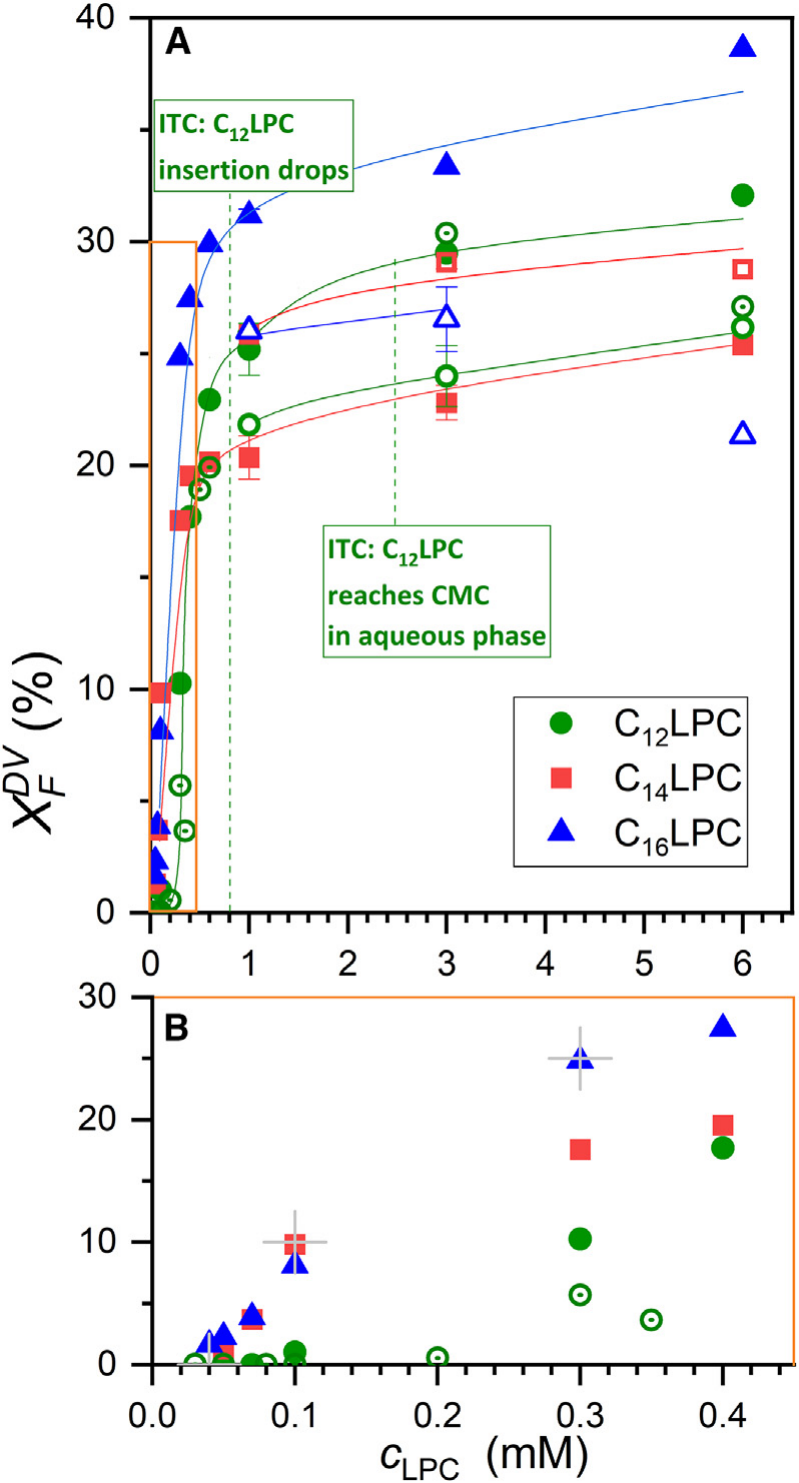
Fraction of fluorescence arising from DVs, X_F_^DV^ of 3.5 mM POPC LUVs after exposure to LPC as a function of LPC concentration. Green spheres represent C_12_LPC, red squares C_14_LPC, and blue triangles C_16_LPC; solid, open, and dot-centered symbols refer to separate liposome batches). Lines guide the eye. Error bars represent the standard deviation. (*B*) Is a zoomed-in window of (*A*) (see *orange frame*). Budding starts at a threshold of about 0.04 mM for C_14_LPC and C_16_LPC and about 0.20–0.26 mM for C_12_LPC, and proceeds up to a limit of the order of 30% approached above ≈1 mM LPC. Conclusions on C_12_LPC from ITC shown in boxes with green text refer to Fan et al. (see main text for reference). Gray crosses denote exemplary points being discussed in the text. To see this figure in color, go online.

While the primary aim of our study is to quantify and explain the budding limit, it is of some interest to have a look at the low-concentration range where budding increases with LPC concentration. Fig. 4 *B* shows a close-up of the region in Fig. 4 *A*. At least for C_14_LPC and C_16_LPC, budding appears to start at about 0.04 mM LPC.

To calculate the membrane composition expected at this point, we need the apparent partition coefficient, often defined on the basis of the mole ratio, *K*:

(1) $$K\equiv \frac{c_{LPC}^{b}}{c_{L}\cdot c_{LPC}^{aq}}$$

with *c*_L_ denoting the lipid concentration (assumed to reside in membranes completely) and *c*_LPC_^b^ and *c*_LPC_^aq^ representing the concentrations of membrane-bound and aqueous LPC, respectively. All concentrations refer to the total sample volume. Taking into account that the total LPC concentration, *c*_LPC_, is just the sum of *c*_LPC_^b^ and *c*_LPC_^aq^, Eq. 1 can be rearranged to:

(2) $$c_{LPC}^{b}=c_{LPC}\frac{K\cdot c_{L}}{1+K\cdot c_{L}}$$

Hoyrup et al. published *K* values of 12 and 93 mM^−1^ for C_14_LPC and C_16_LPC partitioning into DPPC in the fluid phase at 50°C (41). If *K* for POPC at 25°C was at least ≥5 mM^−1^, we find that ≥95% of the LPC is membrane-bound at a lipid concentration of *c*_L_ = 3.5 mM as used here. That means that, at 0.04 mM LPC, the membrane contains about 1 mol % LPC overall, corresponding to ≈2 mol% in the outer leaflet if none flipped to the inner leaflet. For C_12_LPC, the apparent *K* into the unstressed membrane referred to all lipid was measured as 0.3 mM^−1^ (17) so that ≈0.5 of it are membrane-bound according to Eq. 2. The threshold of 0.2–0.26 mM (Fig. 4 *B*) would correspond to *c*_LPC_^b^ ≈ 0.1–0.13 mM and a fraction of 2.8–3.6 mol% C_12_LPC in the membrane. In other words, the weaker partitioning contributes to the higher budding threshold of C_12_LPC seen in Fig. 4 *B*, but may not fully account for it.

Tentatively, we may assign the budding threshold obtained at overall 1 mol% of C_14_LPC or C_16_LPC to an asymmetry between 2 mol% in the outer and 0 mol% in the inner leaflet, i.e., an intrinsic-area asymmetry of ≈2%. It should be noted that this threshold is not dependent on the question of NBD-DSPE distribution between DVs and MVs; at the threshold, there are no DVs yet and, hence, no distribution in the first place.

### Budding power: The progress of budding per lysolipid added

Let us explain the implications of the data at the example of three characteristic points marked with gray crosses in Fig. 4 *B*. From the threshold of budding to first appear at ≈0.04 mM LPC (at 3.5 mM POPC), the fluorescence from DVs increases steeply to about 10% (for C_14_LPC and C_16_LPC) upon increasing LPC to 0.1 mM, i.e., upon increasing the total lipid by only 0.06/3.54 mM = 1.7%. The third cross to be discussed is at 0.3 mM and 25% (essentially a data point for C_16_LPC).

Different thinkable scenarios could account for this finding, including.

i) The fluorescent lipid, NBD-DSPE distributes at a fixed proportion to the POPC, *X*_NBD_^DV^ ≈ *X*_POPC_^DV^, and LPC accumulates in DVs.
ii) NBD-DSPE distributes evenly over all lipid, *X*_NBD_^DV^ ≈ *X*_LIP_^DV^ with LIP standing for the sum of POPC and LPC; accumulation of LPC in DVs is even stronger.
iii) LPC and POPC mix homogeneously over DVs and MVs, but DVs are highly enriched in NBD-DSPE.

A closer look lets us strongly favor assumption (i), that the fraction of fluorescence from DVs represents the budded fraction of POPC. The point at 10% budding at 0.1 mM LPC would then represent the presence of 10%×3.5 mM = 0.35 mM POPC and 0.1–0.04 mM = 0.06 mM LPC in DVs. This corresponds to an average LPC content of the DVs of 15 mol %, arising from 24 mol % within the outer leaflet (assumed to contain 60% of all lipid given its larger radius) and no LPC in the inner leaflet. Retaining just the threshold LPC of 2 mol % in the outer leaflet of MVs, this reflects a more than 10-fold accumulation of LPC in the strongly curved, outer leaflet of DVs. At the third cross in Fig. 4 *B*, *X*_F_^DV^ = 25% at *c*_LPC_ ≈ 0.3 mM, the projected LPC content of the outer leaflet of DVs would be higher, about 38 mol%.

This scenario is, first of all, plausible, considering that the spontaneous curvature of POPC is about zero but that of LPC is strongly positive, so that the energy of the system is relaxed by accumulating LPC in the strongly positively curved outer leaflet of DVs. NBD-DSPE as a two-chain lipid should rather resemble the close-to-zero intrinsic curvature of POPC and distribute accordingly. If NBD-DSPE has a somewhat nonzero intrinsic curvature, the resulting effect on the overall distribution should be attenuated by the fact that it is—in contrast to the LPC—localized in both outer and inner leaflet. A slight enhancement of NBD-DSPE in the outer leaflet of DVs will, thus, be partially compensated for by a depletion in the inner leaflet, and vice versa.

In addition to this plausibility argument, there is some experimental support for the *X*_NBD_^DV^ ≈ *X*_POPC_^DV^ hypothesis. We collected fractions of the AF4 eluate representing the DV and MV regions and measured their fluorescence intensity and lipid (precisely: phosphorus) content outside the AF4 setup. Unfortunately, for the standard setup, the dilution was too strong to obtain measurable phosphorus contents, even with pooling several runs. We, therefore, repeated the AF4 with an overloaded channel, adding 80 *μ*L of sample instead of the standard 5 *μ*L. Given this experimental issue, the results should not be considered conclusive (see Fig. S7 for details), but it is interesting to note that the ratio between fluorescence intensity and phosphorus concentration in the DV fraction was only 70–80% of that in the MV fraction. This would perfectly be in line with the fact that NBD-DSPE distributes along with POPC, which makes 70–80% of the phospholipid in DVs but 98% in MVs.

The second possible assumption (ii) stated above, *X*_NBD_^DV^ ≈ *X*_LIP_^DV^, seems less likely. Experimentally, it would imply the same fluorescence intensity to phospholipid ratio in DVs and MVs, in conflict with our test. Mechanistically, it would require curvature energy contributions to be negligible compared with mixing entropy. If this model was to apply anyway, the fraction of POPC in DVs would be lower than obtained with assumption (i), for example, 20% instead of 25% for the point at about 0.3 mM LPC. The local content of LPC in the outer leaflet of DVs would be 45% instead of 38 mol % for this example. In other words, even if this fundamentally different assumption was correct, despite the arguments speaking against it, the consequences for the interpretation of the data would still be moderate.

The other extreme model (iii) assuming the LPC-POPC-mixture to be the same in DVs and MVs can essentially be ruled out. It would imply an about 10-fold local accumulation of NBD-DSPE in DVs, which does not seem to make sense in terms of curvature energy. Also, the fluorescence intensity to phosphorus ratio in DVs should be 1000% of that in MVs, which should be ruled out by our test, despite its limitations.

Summarizing, we conclude that the fluorescence from DVs versus MVs can be assumed to reflect the distribution of POPC to a good approximation. Accordingly, budding would originally proceed with 5–6 POPC per LPC added, corresponding to a local LPC content of about 24 mol% in the outer leaflet (a more than 10-fold enrichment compared with MVs). As the budded fraction increases, higher LPC contents in the DVs seem to be required so that the number of POPC budded per LPC decreases.

### Budding is limited to about 20–30%

The budding curves in Fig. 4 *A* show a saturation behavior with slopes decreasing toward a common plateau value of the order of 20–30% for all LPC species studied.

Each of the data sets shown in Fig. 4 has been produced with an individually prepared batch of POPC LUVs. Whereas all curves show a rather smooth saturation behavior (except for outliers at 6 mM C_16_LPC and C_12_LPC), there is some batch-to-batch variability of the budding limit. This suggests that details of the extrusion or, generally, vesicle preparation procedure of a given batch have some effect on the budding limit. This finding supports the hypothesis that the budding limit is, in fact, controlled by the “sphericity,” i.e., the area-to-volume ratio of the original vesicles. This hypothesis will be challenged below. In contrast to what one might have expected, the chain length (and, hence, CMC, partition coefficient, intrinsic curvature, etc.) of the LPC has no marked effect on the measured budding limit (height of the *X*_F_^DV^ plateau) that would be detectable despite the batch-to-batch variation.

The state of the samples with increasing concentration of C_12_LPC (*green data points* in Fig. 4 *A*) can be understood on the basis of isothermal titration calorimetry (ITC) data titrating C_12_LPC into 3.5 mM POPC LUVs (see Fig. 1 *B* of Fan et al; (17)). Up to about 0.7 mM C_12_LPC, the injected micelles dissolve and the LPC partitions into liposomes. As indicated by the data in Fig. 4, this membrane insertion is facilitated by progressive budding, keeping asymmetry stress tolerably low. At about 0.7 mM C_12_LPC, ITC indicates a strong drop of membrane insertion so that now, added C_12_LPC remains in solution and the heat of injection becomes negative. The present data (Fig. 4 *A*) link this insertion limit seen by ITC to a budding limit. Budding occurs to partially release asymmetry stress in the membrane caused by uptake of C_12_LPC into the membrane and, apparently, is a prerequisite for the membrane insertion of further LPC.

Only at about 2.5 mM C_12_LPC, the heats detected by ITC vanish, indicating that the aqueous concentration of C_12_LPC has reached its CMC and micelles coexist with vesicles having reached their (kinetic) limit of C_12_LPC uptake. This appearance of micelles has no detectable effect on budding (see *second green box* in Fig. 4 *A*), which remains at the limit reached already at much lower C_12_LPC concentration.

### The model of conserved area, volume, and asymmetry

It has been recognized before that budding, which ideally proceeds without leakage or lipid scrambling (meaning conserved asymmetry), has to maintain both the overall membrane area, *A*, as well as the sum of the interior volumes, *V*, of the vesicles (25,52,53). Stretching a membrane in area is opposed by a substantial stretching modulus of 243 mN×m^−1^ (54). A reduction in volume could theoretically be achieved by the efflux of water but in the presence of salt or other membrane-impermeant solutes, this is strongly opposed by the osmotic pressure it generates.

This constraint of conserved area and volume cannot be met starting with a sphere, given the size-dependent area-to-volume ratio. For example, splitting one sphere of 100 nm diameter into two of ≈80 nm keeps the volume constant but requires a 1.25-fold larger surface area. That means, for a splitting of a vesicle to occur, it would need to start with a nonspherical one with an excess area, *A*_E_, that makes up for the additional area requirement. The excess area of a vesicle of any unknown shape is defined as the difference between its true surface area, *A*, and the surface area of an ideal sphere with the same volume, *A*_sph_(*V*):

(3) $$A_{E}=A-A_{sph}\left(V\right)=A-3V^{2/3}$$

Hence, the simplest model (conserved *A*, *V*, asymmetry) implies that an asymmetry stress causes recurrent budding until either the stress is relaxed below a budding threshold (about 2% area asymmetry according to Fig. 4 *B*) or until the MVs has become spherical, *A*_E_ ≈ 0. Note that an excess area allows for fluctuations of the membrane that are entropically favored so that the limit is not kicking in suddenly at *A*_E_ reaching zero but a reduction in *A*_E_ will increasingly be opposed. This may account for the more gradual saturation of the budding curves (Fig. 4) as opposed to a straight line all the way from threshold to limit as suggested for an ideal pseudo-equilibrium between MVs and DVs of fixed internal compositions. The occurrence of nonspherical vesicles after extrusion is known and can be observed by cryo-TEM images (55,56,57). The shape and thus the trapped volume in the extruded vesicle are controlled by the passage through the filter (57).

### The budding limit correlates with the excess area of the vesicles

Interestingly, Eq. 3 offers a simple method to adjust the excess area of a vesicle. Exposing the vesicle, for example, to a hyperosmotic environment of twice the salt concentration will cause a water efflux. This deflates the vesicle so that *V* becomes half the original value and *A*_E_ increases accordingly. For this case, the model predicts a higher budding limit. Exposing vesicles to a hypoosmotic exterior will inflate them, and reduce *A*_E_ and the budding limit. If the exterior is still strongly hypoosmotic as the inflated vesicles get spherical, the latter might burst.

**Figure 5:**
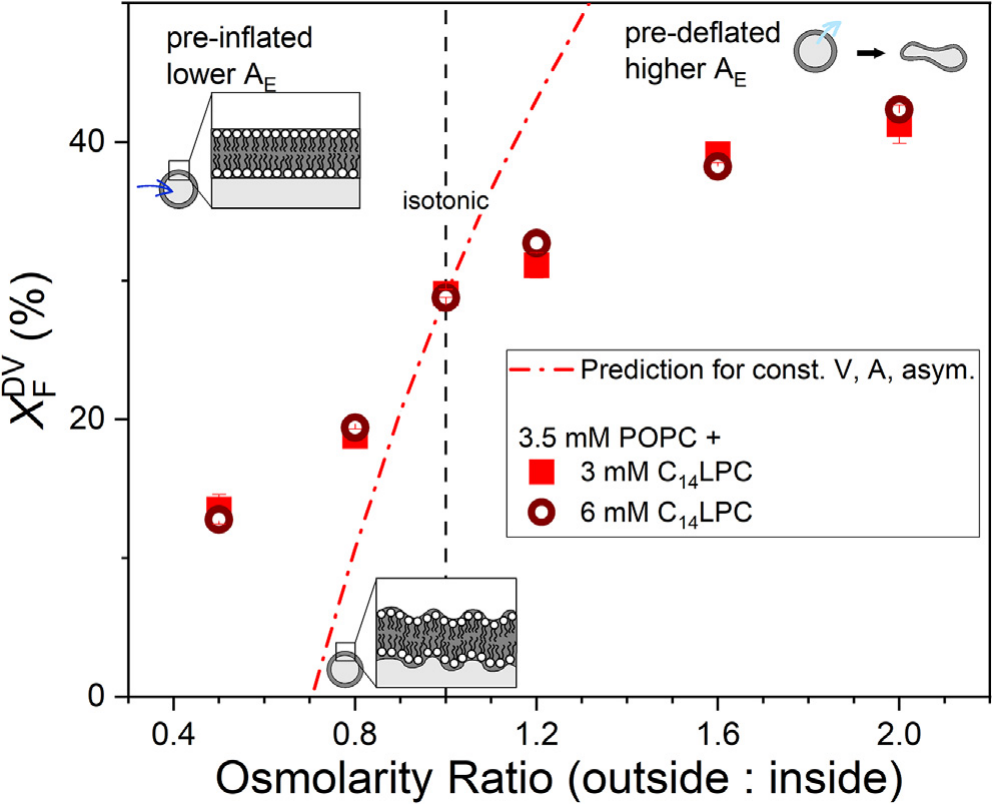
Budded fraction of fluorescence arising from DVs, X_F_^DV^ in dependence of the osmolarity ratio of the POPC LUVs to the outside buffer that have been applied before incubation with C_14_LPC (*red symbols*). Error bars represent the standard deviation. The amount of C_14_LPC is sufficient to reach the plateau of the budded fraction (see Fig. 4*A*). By inflating (hypotonic buffer) or deflating (hypertonic buffer) the vesicles, the excess area of the vesicles is lower (hypotonic) or higher (hypertonic) compared with isotonic conditions, which is presumably a limiting factor to the vesicle budding process. The calculated fraction of fluorescence in DVs using a model of conserved volume, area, and asymmetry confirms the dependence qualitatively but not quantitatively. To see this figure in color, go online.

The corresponding experiments were performed by extruding vesicles in standard buffer (initial osmolarity) and subsequently changing the osmolarity of the outside buffer (final osmolarity). As long as *A*_E_ > 0, the ratio between final and initial osmolarity should cause a proportional change in *V* (58). *A*_E_ should, then, increase with final osmolarity in a continuous yet nonlinear fashion. In a next step, these predeflated or preinflated vesicles were exposed to LPC to induce limiting budding (Fig. 5). The agreement of the budding fractions obtained with 3 and 6 mM LPC confirms that the budding limit has been reached. The primary implication of Fig. 5 is that the budding limit increases with excess area, as predicted by the model.

The hyperosmotic environment leads to higher budded fraction while hypotonic medium decreases the budded fraction compared with isosmotic conditions. The greater the excess area until it reaches a perfect sphere, the higher the budded fraction and vice versa. This strongly suggests that membrane smoothing is a limiting factor to the budding process.

### Quantitative assessment using the model of conserved volume, area, and asymmetry

In the supporting material, a calculation is presented that quantifies the tonicity-dependent change of the budding limit that would be predicted in the ideal case, that osmotic treatment 1) does not affect membrane area and 2) changes internal volume exactly to the extent that osmolality between interior and exterior match. Furthermore, 3) budding is assumed to proceed with the same extent and kinetics until the MVs become ideally spherical; then budding stops at once. The calculated, ideal trace (see *red dash-dotted line* in Fig. 5) represents a stronger effect of osmotic treatment than found experimentally. A number of limitations and simplifications of the model is likely to account for this difference. Particularly at low salt, the entrapped volume may be reduced (i.e., the budding limit increased) by water efflux via diffusion (low absolute osmotic pressures) or vesicle rupture. Particularly at higher budding limits, a partial loss of asymmetry by a transient flip of lipids that seems plausible to accompany each fission event may reduce real budding compared with the model prediction.

Table 1 shows a compilation of parameters derived here and taken from the literature.

**Table 1:** Compilation of parameters derived here and taken from the literature

	C_12_ LPC	C_14_LPC	C_16_LPC
Budding threshold: *X*_LPC_^MV,out^ at onset	2–4 mol%	2 mol%	2 mol%
Budding power: *n*_POPC_/*n*_LPC_ in DVs	3.5–5	3.5–5	3.5–5
Budding limit: *X*_F_^DV,max^ at 3.5 mM POPC, isotonic	22–37%	22–37%	22–37%
r of daughter vesicles (MALS data) (nm)	16 ± 6	16 ± 6	16 ± 6
r of mother vesicles (MALS data) (nm)	46 ± 1	46 ± 1	46 ± 1
K (mM^−1^)	0.3 (17)	12 (41)	93 (41)
CMC (*μ*M)	560 ± 50 (17)	45 ± 2 (40)	4 (40)

### Attempting to detect asymmetry strain

Asymmetry stress causes a strain that compresses the molecular area and increases the order on the outer leaflet and/or expands molecular area and decreases order in the inner leaflet as illustrated by Fig. 1. On the basis of published ITC data (17) and the budding results shown above, we have hypothesized that it needs only very little stress to start budding. As long as budding is possible, it can be expected to avoid any significant asymmetry strain to be built up. As the budding limit is reached at about 0.7 mM LPC added to 3.5 mM POPC, this pathway of stress relaxation is not available any further. Further addition of LPC should increase the stress and resulting strain, until this stress inhibits further LPC insertion into the membrane (“staying out”).

Our strategy to check for asymmetry strain uses the time-resolved fluorescence anisotropy of TMA-DPH. Given its trimethyl ammonium moiety, the probe is attached to a membrane surface and does not spontaneously flip between leaflets during the time needed to finish the experiments (59). The higher its limiting anisotropy, *r∞*, the more restricted is its angular motion within the lipid leaflet, meaning the higher is the order of the latter (60). We have compared *r∞* of TMA-DPH located symmetrically in both leaflets with that of TMA-DPH added to preformed vesicles, i.e., expected to be located exclusively in the outer leaflet. Unfortunately, there is no established protocol to insert TMA-DPH exclusively into the inner leaflet but assuming that the symmetrically distributed probe reflects essentially the average of the order in the outer and inner leaflet; comparing data from outer and from both leaflets also provides information about the inner leaflet.

It must be emphasized that this assay remains to be established and challenged in much more detail to render the interpretation in terms of asymmetry strain compelling. That effort is outside the scope of this paper. Nevertheless, we have decided to present the respective data here; more on this method is to follow.

**Figure 6:**
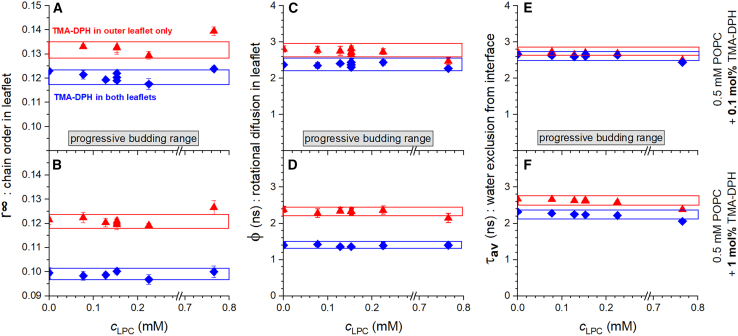
Limiting fluorescence anisotropy (extrapolated to infinite time after excitation), r∞ (*A* and *B*), rotational correlation time, φ (*C* and *D*), and average lifetime, τ_av_ (*E* and *F*), as a function of the concentration of C_12_LPC (c_LPC_) added to samples containing 0.5 mM POPC and either 0.1 mol % (upper panels *A*, *C*, and *E*) or 1 mol % (lower panels *B*, *D*, and *F*) of TMA-DPH. The probe, TMA-DPH was either mixed with the lipid in organic solution and, hence, distributed homogeneously over outer and inner lipid leaflet (*blue diamonds*) or added after vesicle formation and, hence, present exclusively in the outer leaflet (*red up triangles*). Error bars represent standard errors of the fit of the anisotropy decay. Gray boxes indicate the concentration range in which progressive budding is expected, i.e., from budding threshold to budding limit. Blue and red boxes are to guide the eye. To see this figure in color, go online.

Different batches of samples with different TMA-DPH contents in POPC in symmetrical or asymmetrical insertion in LPC-free vesicles gave rise to limiting anisotropies of about 0.1–0.13 at 25°C (Fig. 6). This range also matches the batch-to-batch variation of five supposed-to-be-identical samples produced in our laboratory by different persons using individual protocols and batches of lipid, suggesting that these differences might not truly represent effects of local concentration or asymmetry. This variability may also explain counterintuitive differences of related literature data. Although *r∞* is expected to decrease with increasing temperature, 0.10–0.11 were reported for POPC at 20°C (60), 0.18 at 23°C (61), and 0.17 for 30°C (62).

Despite this variability of absolute values, *r∞* has been proven useful to monitor order changes within one batch upon addition of perturbants. Increasing membrane contents of typical detergents caused a progressive disordering down to a characteristic value of about 0.010 for DPH and about 0.083 for TMA-DPH (20°C), when the membrane became disintegrated to mixed micelles (60).

The *r∞* data appear to be constant (or slightly decreasing; see *boxes* in Fig. 6) throughout the concentration range where budding proceeds. Slightly beyond the budding limit, however, the order revealed by the outside-only TMA-DPH is higher (outside the box) for both probe concentrations. This is what one should expect if the assay works as hypothesized and if substantial asymmetry strain builds up only after the budding limit has been reached. This would be well in line with our interpretation of the budding data.

No significant changes throughout the budding range were also found for the dynamic parameters of TMA-DPH, the rotational correlation time, φ (Fig. 6, *C* and *D*), and the amplitude-averaged fluorescence lifetime, *τ* (Fig. 6, *E* and *F*). With all due caution of interpreting the lack of an effect and acknowledging the complexity of the systems comprising MVs, DVs, and, at high LPC, potentially micelles, this is an interesting finding. Typically, increasing strains tend to speed up rotation (decrease φ) and increase water accessibility of the probe (decrease *τ*) (60). Hence, the lack of any detectable effect within progressive budding range is in line with the idea proposed here that budding accommodates all bilayer (and monolayer) curvature stress and avoids any substantial strains. Beyond the budding limit, slight drops of φ and *τ*, in particular for the outside-only probe, are compatible with some strains building up.

## Conclusion

AF4 was successfully established to quantify the extent of budding of LUVs induced by the addition of LPCs.

At physiological salt concentration, budding starts at a threshold of roughly 2% intrinsic area asymmetry and proceeds with about 3.5–5 POPC molecules budding with one C_14_LPC or C_16_LPC. It reaches a plateau after budding 20–30% of the fluorescence probe into DVs, the “budding limit.” The fraction of fluorescence from DVs can likely be interpreted in terms of the budded fraction of the membrane lipid, POPC.

This limit does not depend substantially on the chain length of the LPC (for C_12_–C_16_) but varies between different vesicle preparations, likely depending on the sphericity (precisely: the excess area) of vesicles reached upon extrusion.

The hypothesis that this budding limit is governed by the excess area of the initial liposomes was supported by experiments with predeflated and preinflated vesicles. Accordingly, budding “uses up” excess area and must stop as the MVs reaches a fully spherical shape (zero excess area). A very basic model was derived assuming constant volume, area, and asymmetry of the liposomes and the absence of alternative relaxation phenomena. It overestimated the osmotic effects on the budding limit, indicating that these assumptions were not strictly met in the experiments.

We propose that budding starts at a low stress threshold (activation energy) and allows for avoiding the buildup of significant curvature stresses, both in the bilayer and within the monolayer. This renders it the primary response of bilayers to the asymmetric insertion of impermeant, surfactant-like molecules. Another response mechanism (staying out, micellar solubilization, cracking in, etc.) kicks in only as budding reaches its limit. The results are in accord with what has been reported before for GUVs. Studying budding from LUVs as established here is challenging but permits the quantification of budding at precisely known lipid concentration.

## Author contributions

H.H. conceived the study. L.H. designed, performed, and evaluated the AF4 experiments. M.K. developed the AF4 method. I.C. designed, performed, and evaluated the time-resolved anisotropy experiments. Cryo-TEM experiments were designed and evaluated by A.M. and carried out by G.H. L.H. wrote the first draft with input from other authors. L.H., H.H., and all other authors rendered the manuscript to its final form.

## Supporting citations

Reference (63) appears in the supporting material.

## Supporting material

Document S1. Figures S1–S7 and Tables S1 and S2

Document S2. Article plus supporting material
