# Supplementary material for: Vesicle budding caused by lysolipid-induced asymmetry stress
Source: Biophys J. 2023 Aug 29;122(20):4011–22. doi: 10.1016/j.bpj.2023.08.023 (PMC10598287; doi:10.1016/j.bpj.2023.08.023)
Supplement: Document S1. Figures S1–S7 and Tables S1 and S2 [file mmc1.pdf]

Biophysical Journal, Volume *122*

Supplemental information

Vesicle budding caused by lysolipid-induced asymmetry stress

Lisa Hua, Michael Kaiser, Iulia Carabadjac, Annette Meister, Gerd Hause, and Heiko Heerklotz

Supporting Information

Vesicle Budding caused by Lysolipid-Induced Asymmetry Stress

Lisa Hua[1]*, Michael Kaiser[1], Iulia Carabadjac[1], Annette Meister[2], Gerd Hause[3], Heiko Heerklotz[1,4,5]*

1 Institute of Pharmaceutical Sciences, University of Freiburg, Freiburg, Germany
2 ZIK HALOmem and Institute of Biochemistry and Biotechnology, MLU Halle-Wittenberg, Halle, Germany
3 Biozentrum, MLU Halle-Wittenberg, Halle, Germany
4 Leslie Dan Faculty of Pharmacy, University of Toronto, Toronto, Canada
5 Signaling Research Center BIOSS, University of Freiburg, Freiburg, Germany

*Corresponding authors: lisa.hua@pharmazie.uni-freiburg.de; heiko.heerklotz@pharmazie.uni-freiburg.de

Contents

AF4 elution profile 2
Quantification of budded fraction by fluorescence 2
Reference Pictures Cryo-TEM 4
Total fluorescence integral of AF4 chromatograms 4
Vesicle budding proceeds without significant contents leakage 5
The budded fraction is independent of the incubation time (2 min - 11 h) 6
Distribution of the fluorescence probe in daughter and mother vesicles 7
Model of conserved area, volume and asymmetry 8
Supporting References 9

AF4 elution profile

The eluent was always of the same osmotic concentration as the injected sample. A channel flow of 1.0 mL·mL^{-1} and an inject flow of 0.2 mL·mL^{-1} was used. A detailed flow profile is displayed in Table S1.

Table S1: Timetable of AF4 elution profile; a channel flow of 1.0 mL·mL^{-1} and an inject flow of 0.2 mL·mL^{-1} was used.

**Mode**	**Duration (min)**	**Crossflow Start (mL·mL^{-1})**	**Crossflow Stop (mL·mL^{-1})**	**Flow Profile**
Elution	2.0	0.80	0.80	Constant
Focus	2.0	0.80	0.80	Constant
Focus Inject	4.0	0.80	0.80	Constant
Focus	4.0	0.80	0.80	Constant
Elution	20.0	0.80	0.80	Constant
Elution	23.0	0.80	0.05	Linear
Elution	15.0	0.05	0.05	Constant
Elution Inject	5.0	0.00	0.00	Constant
Focus	2.0	0.80	0.80	Constant
Elution	2.0	0.80	0.80	Constant

Quantification of budded fraction by fluorescence

During separation using the AF4, baseline separation of daughter vesicle and mother vesicle peaks could not be achieved. We attribute this also to the re-fusion of daughter vesicles during passage across the separation channel of the AF4. As eluent lacks C_{14}LPC compared to *in situ* conditions and the partitioning of LPC into a POPC bilayer is a dynamic (reversible) process, some LPC is extracted. This extraction results in instability of daughter vesicles, subsequent refusion and a population of particles between daughter vesicles and mother vesicles.

To quantify *in situ* fraction of lipid present in the form of daughter vesicles, the following method was developed. For this, three assumptions were made:

1. The peak shape of mother vesicles and the initial vesicles are nearly identical. A reasonable assumption as initial vesicles are of similar size and narrow size distribution and therefore should have the same capacity for budding. That is, each vesicle should change in a similar fashion to all other vesicles, resulting in a shift of the peaks location, but no change in shape.
2. The observed population between daughter vesicles and mother vesicles is from re-fused daughter vesicles and not from for example mixed micelles.
3. The contribution of re-fused species in the size range of mother vesicles is negligible. POPC vesicles become (geometrically) stable at a radius much smaller than the ~ 50 nm of mother vesicles.

If these assumptions hold, a reference chromatogram of initial vesicles must closely resemble the peak shape of the mother vesicle and the remainder – obtained from normalization against peak height followed by subtraction – yields the contribution to fluorescence by daughter vesicles and their re-fused species. The subtraction of the chromatograms is displayed in Figure S1.

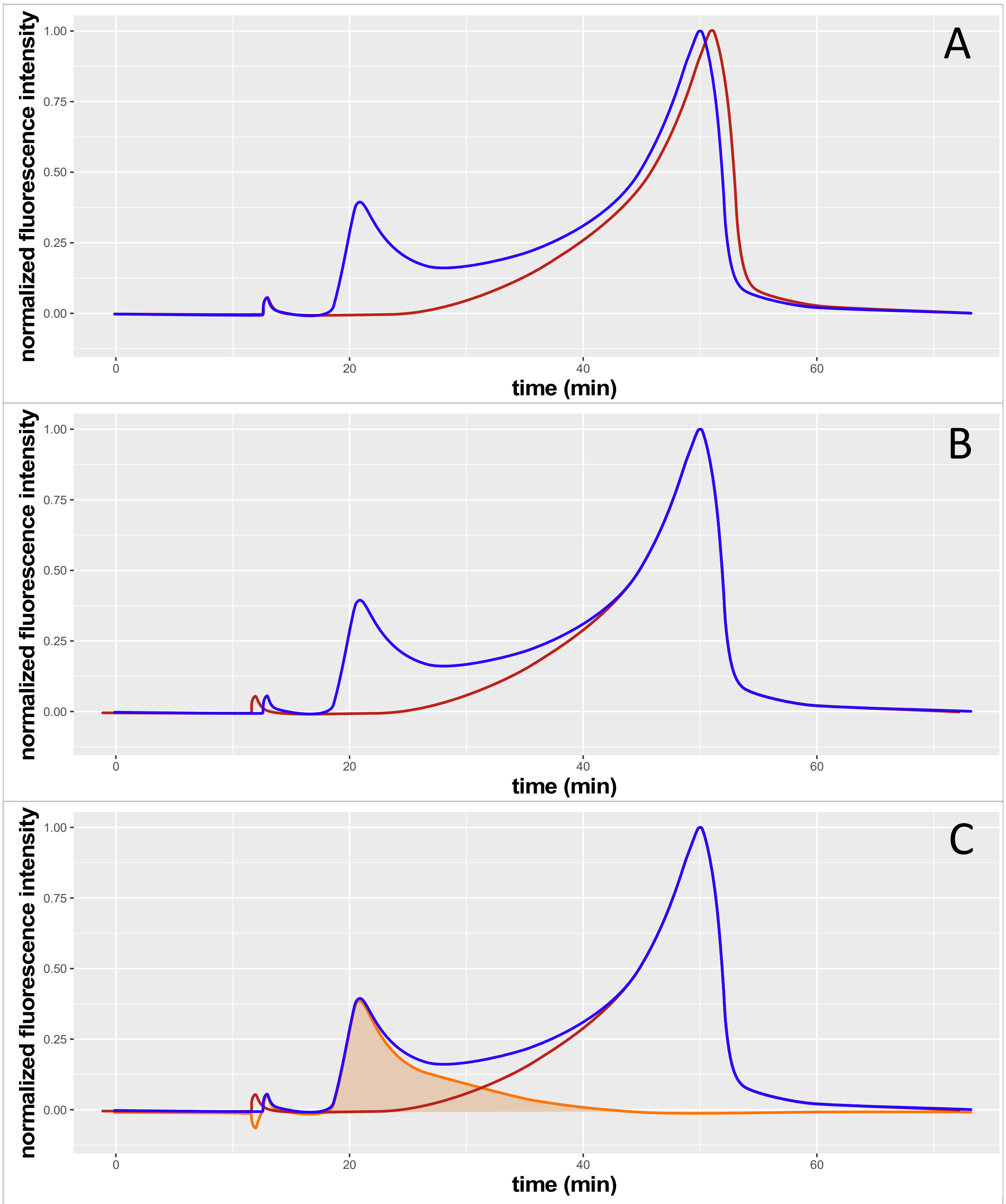

*Figure S1: Fluorescence chromatograms obtained after separation by AF4; shown are the steps in their transformation, yielding "budded fraction of fluorescence". **A)** Chromatogram of initial vesicles (red) and daughter vesicles + mother vesicles (blue); **B)** the two chromatograms are aligned on the peak-maxima; **C)** given certain assumptions, subtraction of the chromatogram of the initial vesicles from daughter vesicles + mother vesicles will yield the contribution of fluorescence from daughter vesicles and refused species.*

Reference pictures Cryo-TEM

Figure S2A shows that without addition of LPC, no daughter vesicles are visible. Therefore, the addition of LPC is a mandatory prerequisite to obtain the smaller daughter vesicles. Panel B show the same sample after heating up to 65 °C for 1 h. Without the addition of LPC, no solubilization after heating up the sample is visible.

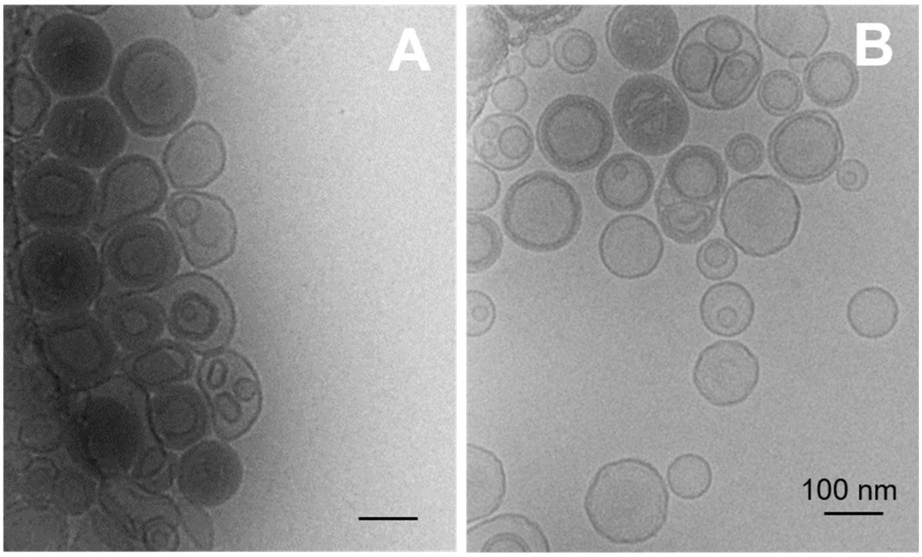

Figure S2: Cryo-TEM images of a sample of 2 mM POPC LUVs. Panel A shows vesicles prepared at room temperature. Panel B shows the same sample after a heating it up to 65°C for 1 h. The vesicles remain intact.

Total fluorescence integral of AF4 chromatograms

Figure S3 show the total fluorescence integral of the AF4 chromatograms of two different liposome preparation batches. The overall total fluorescence integral is stable within one liposome batch and does not differ substantially with addition of LPC. This indicates the reproducibility of the presented AF4 experiments.

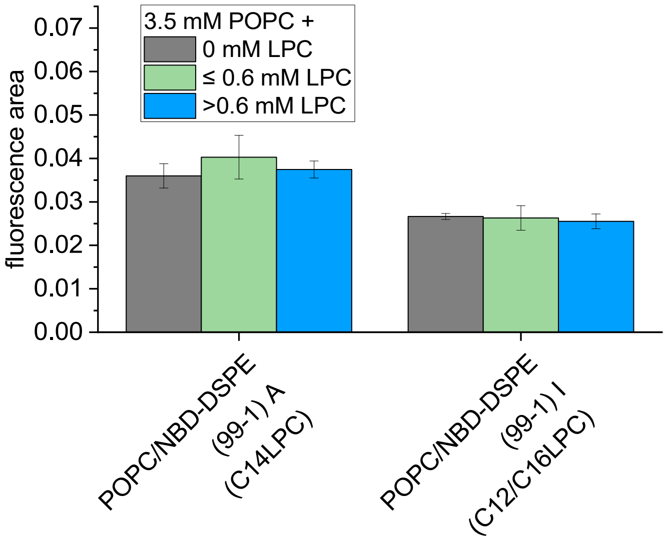

Figure S3: Total fluorescence integral of AF4 chromatograms. Displayed are the runs with at least three runs in all sections (no LPC, before and after the plateau threshold (0.6 mM LPC)) at isotonic conditions to have a representative sample for each bar. The two sets represent two different liposome preparation batches of POPC + 1 mol% NBD-DSPE LUVs.

Vesicle budding proceeds without significant contents leakage

Leakage assays were performed with calcein-loaded POPC LUVs with varying concentrations of LPC with a protocol established by Patel et al. (1). Figure S4 show a time-dependent measurement with C_{14}LPC. Keeping in mind that the AF4 experiments are usually done with an incubation time of only two minutes, the amount of total leakage is very minimal. This holds even for 24 h of incubation. 6 % of leakage upon incorporation with the highest LPC concentration, 67 µM, for 24 h might indicate the slow progress of micellar solubilization.

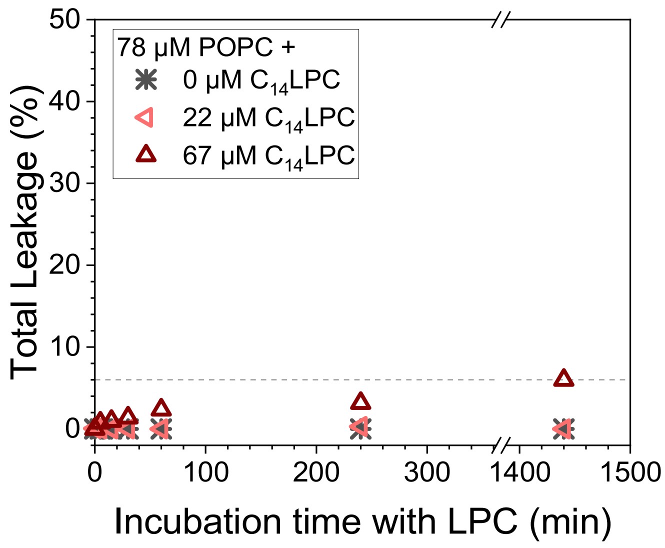

Figure S4: Time dependent leakage results with POPC LUVs with varying C_{14}LPC Concentrations. In the observed LPC concentrations nearly no leakage is detectable even after an incubation time of 24 hours.

Figure S5 show that no substantial leakage is detectable with varying concentration of C_{12}, C_{14} and C_{16}LPC. The highest concentration used in the experiments is enough to trigger vesicle budding in the given conditions. For C_{14} and C_{16}LPC, it is even expected that the budding plateau is already reached under these conditions. However, no leakage is detectable.

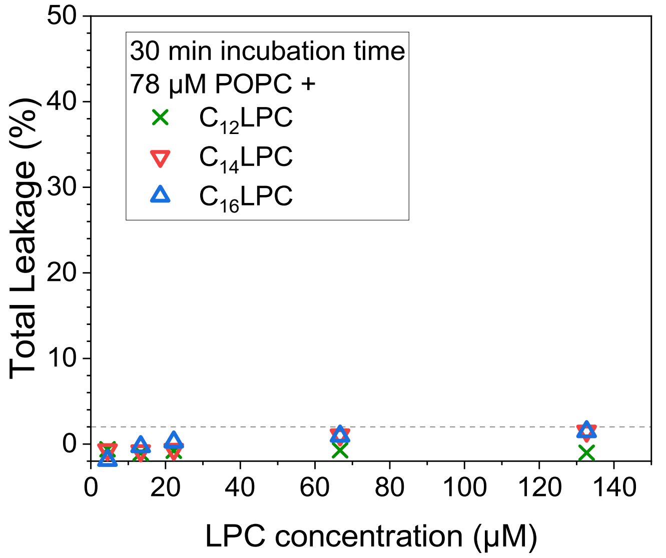

Figure S5: Leakage measurements with 78 µM POPC LUVs in addition of varying concentrations of LPC with C_{12}, C_{14} or C_{16} acyl chains with an incubation time of 30 min.

The budded fraction is independent of the incubation time (2 min - 11 h)

Figure S6 shows that the budded fraction of a sample reaches a steady state within the standard incubation time of 2 min. Long incubation times up to 11 h do not result in stronger budding. This indicates that no quantitative flipping of LPC occurs during the duration. Budding proceeds quickly and to completion. Also, daughter vesicles remain stable and do not fuse in the given time frame.

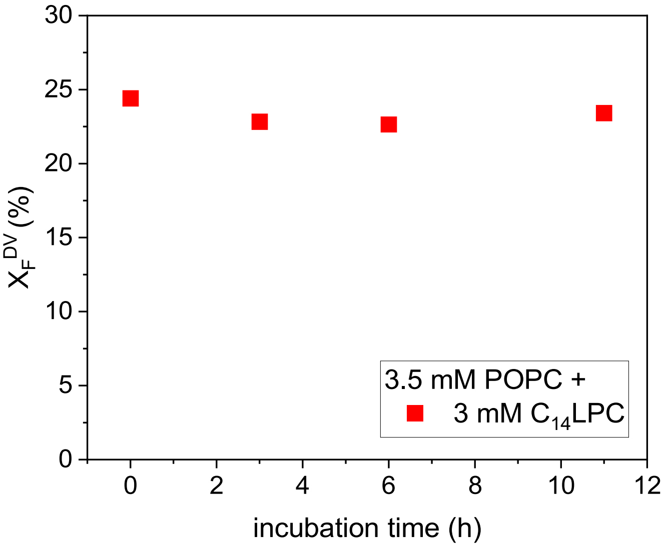

Figure S6: Time dependent budded fraction of fluorescence, X_F^{DV} of a sample with 3.5 mM POPC vesicles (with 1 mol% NBD-DSPE) incubated with 3 mM C_{14}LPC. X_F^{DV} remains stable after at least 11 h of incubation with LPC.

Distribution of the fluorescence probe in daughter and mother vesicles

To determine the distribution of fluorescence probe between DV and MV, samples of the respective fractions were collected after the separation on the AF4 channel to determine their fluorescence intensity and lipid (i.e., phosphate) concentration.

Because the sample is heavily diluted during AF4 separation, it was needed to increase the injection volume of the sample from the standard value of 5 µL to 80 µL per run and to pool respective fractions of two separate runs.

A chromatogram of such an "overloaded" run is shown as Figure S7, showing the characteristic peaks previously assigned to DV and MV plus an additional one at an early elution times of about 18 min. Changes of this peak as a function of focusing time (not shown) suggest it to represent some amount of material that was eluted without proper focusing and, hence, separation. Whereas this jeopardizes the quantitative evaluation of the subsequent profile, it seems well separated and should not alter the qualitative composition of the DV and MV fractions following.

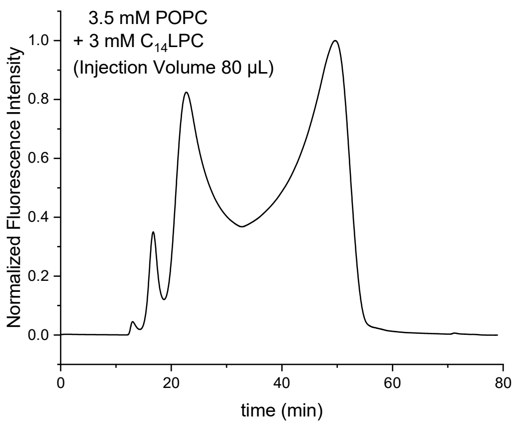

Fig. S7: Fluorescence intensity at a function of elution time after injecting 80 µL of a sample of 3.5 mM POPC + 1 mol% NBD-DSPE and 3 mM C_{14}LPC into the AF4 system after 2 min of incubation. Note that the injection volume is higher than recommended.

A steady-state fluorescence emission spectrum was recorded for the samples for mother and daughter vesicles. Measurements were executed with the high-performance spectrometer FluoTime 300 (PicoQuant, DE) in polystyrene cuvettes (Sarstedt, DE) cuvettes at 25 °C under continuously stirring. Excitation was performed at the wavelength of 465 nm. Emission was recorded through a 470 nm filter from 470 nm to 670 nm. Afterwards, a Bartlett-Assay (2) was performed with the same sample to determine the phosphate concentration that includes the sum of the LPC as well as the POPC and NBD-DSPE concentration. The fluorescence intensity at the spectrum's maximum (at 520 nm) was then correlated to the total phosphate concentration of the sample.

If the results are correct in spite of the high injection volume, they support the idea that the fluorescence scales with the POPC content of the fractions, given that DV typically contain 70 – 80 mol% of POPC (see main text) and a similar amount of fluorescence per total lipid, compared to MV (Table S2).

Tab. S2: Results of analyzing daughter vesicle (DV) and mother vesicle (MV) fractions of two AF4 runs with respect to their phospholipid concentration (phosphate assay) and integrated fluorescence intensity. 3.5 mM of POPC + 1 mol% NBD-DSPE were incubated with 3 mM C_{14}LPC for 2 minutes and 80 µL (more than the standard amount of 5 µL) were injected into the AF4. The results have to be considered with caution since the injection volume had to be chosen significantly larger than standard in order to obtain a measurable lipid concentration after elution.

Batch	Fraction	Phosphate concentration (µM)	Fluorescence intensity (counts)	Fluorescence per phosphate (counts/µM)	Fluorescence per phosphate, normalized to MV
1	DV	8.5	5863	692	**79%**
	MV	13.3	11666	880	100%
2	DV	7.8	4884	626	**72%**
	MV	12.8	11159	870	100%

Model of conserved area, volume and asymmetry

Quantitative predictions of budded fraction in non-isotonic conditions: model of conserved area, volume and asymmetry

One prerequisite of the model is that the excess surface area that is stored in the undulations in the initial (aspherical) vesicle (subscript "0"). By budding off little daughter vesicles (subscript "DV") induced by LPC this excess surface area is removed. The budding process stops when the mother vesicle reaches an ideally spherical shape (subscript "IS"). A model is given here to evaluate the accuracy of the constant volume, area and asymmetry assumption throughout the budding process. A close match in predicted and observed X_F^{DV} will then suggests that membrane smoothing is based on sole geometric assumptions is a dominating limiting mechanism.

The experimental data gives access to following parameters:

- Intensity-weighted geometric radius of daughter vesicles: $r_{\mathrm{z,DV}}$
- Intensity-weighted geometric radius of ideal spherical mother vesicles: $r_{\mathrm{z,IS}}$
- Budded fraction of fluorescence probe in daughter vesicles: X_F^{DV}

Since daughter vesicles and the final mother vesicles are presumably spherical, geometric equations describing spheres are used to calculate internal volume and surface area. Also, a bilayer thickness x_{POPC} of 4.2 nm for POPC membranes (3) was included to account for the loss of volume due to the non-zero bilayer thickness.

Based on two very simple assumptions we established a predictive model to use the results at isotonic conditions to determine the budded fraction at non-isotonic conditions. First, we assume that during the budding process the total surface area A_0 is only redistributed. That means that the total surface area remains constant.

$$A_0 = \frac{A_{IS}}{1 - X_F^{DV}} \tag{1}$$

Second, the internal volume V_0 of the vesicles remains constant, assuming that no significant leakage happens during the budding process.

$$V_0 = V_{IS} + n_{DV} \cdot V_{DV} \tag{2}$$

At isotonic conditions one would obtain a certain budded fraction for an initial total volume and surface area. This budded fraction is assigned to an initial internal volume at isotonic conditions.

By changing the osmolarity ratio of the internal and outer medium, the initial internal volume V_0 is tuned to obtain:

$$V_{tuned} = V_0 \cdot \frac{c_{iso}}{c_{tuned}} \tag{3}$$

The total surface area A_0 is unaffected by the change in tonicity.

$$A_{tuned} = A_0 \tag{4}$$

At hypotonic conditions the internal volume increases. Therefore, the excess surface area that can be removed is decreasing and thus, the predicted budded fraction decreases. At hypertonic conditions the opposite happens with a decrease of internal volume due to water outflux. Hence, more excess surface area is available for the budding process and X_F^{DV} increases.

The tuned volume and total surface area can then be obtained by calculating them from the radius of the ideally spherical mother vesicles as well as the radius and number of daughter vesicles– both obtained experimentally. By using eq. 3 and eq. 4 and assuming that the size of the daughter vesicles is independent on tonicity and while the number of daughter vesicles varies, we may obtain numerical predictions (i.e. the red dash-dotted line in Figure 5). Both, the tuned surface area as well as the volume, has two variable parameters in the equation.

$$A_{\mathrm{tuned}\,,calc} = A_{IS} + A_{DV} = 4\pi \cdot \left(r_{IS} - \frac{x_{POPC}}{2}\right)^2 + n_{DV} \cdot A_{DV} \tag{5}$$

$$V_{\mathrm{tuned},\,calc} = V_{IS} + V_{DV} = \frac{4\pi}{3} \cdot \left(r_{IS} - \frac{x_{POPC}}{2}\right)^3 + n_{DV} \cdot V_{DV} \tag{6}$$

A numerical solution was found using the Excel Solver. For this, number of daughter vesicles and radius of the ideally spherical mother vesicle (above marked with green color) was varied with the aim to minimize the squared difference of V_{tuned} and A_{tuned}. With the found solution for r_{IS} and n_{DV} the budded fraction can be calculated with eq. 1.

Supporting References

1. Patel, H., C. Tscheka, and H. Heerklotz. 2009. Characterizing vesicle leakage by fluorescence lifetime measurements. *Soft Matter*. 5:2849–2851.

2. Bartlett, G.R. 1959. Phosphorus Assay in Column Chromatography. *J. Biol. Chem.* 234:466–468.

3. Pinisetty, D., D. Moldovan, and R. Devireddy. 2006. The effect of methanol on lipid bilayers: An atomistic investigation. *Ann. Biomed. Eng.* 34:1442–1451.
